# Improved Performance of Newborn Screening for Congenital Adrenal Hyperplasia Using 21-deoxycortisol Measurement

**DOI:** 10.1210/jendso/bvaf157

**Published:** 2025-10-09

**Authors:** Sarah E Lawrence, Janet Marcadier, Sheila Auger, Erika Bariciak, Pranesh Chakraborty, Amy Chambers, Susanne Eagle, Michael Kowalski, Alexa Marr, Nathan McIntosh, David S Saleh, Emeril Santander, Graham Sinclair, Diane K Wherrett, Matthew P A Henderson

**Affiliations:** Department of Pediatrics, Children's Hospital of Eastern Ontario, 401 Smyth Road, Ottawa, ON, Canada K1H 8L1; Department of Pediatrics, University of Ottawa, 75 Laurier Avenue East, Ottawa, ON, Canada K1N 6N5; Newborn Screening Ontario, 415 Smyth Road, Ottawa, ON, Canada K1H 8M8; Newborn Screening Ontario, 415 Smyth Road, Ottawa, ON, Canada K1H 8M8; Department of Pediatrics, Children's Hospital of Eastern Ontario, 401 Smyth Road, Ottawa, ON, Canada K1H 8L1; Department of Pediatrics, University of Ottawa, 75 Laurier Avenue East, Ottawa, ON, Canada K1N 6N5; Newborn Screening Ontario, 415 Smyth Road, Ottawa, ON, Canada K1H 8M8; Department of Pediatrics, Children's Hospital of Eastern Ontario, 401 Smyth Road, Ottawa, ON, Canada K1H 8L1; Department of Pediatrics, University of Ottawa, 75 Laurier Avenue East, Ottawa, ON, Canada K1N 6N5; Newborn Screening Ontario, 415 Smyth Road, Ottawa, ON, Canada K1H 8M8; Newborn Screening Ontario, 415 Smyth Road, Ottawa, ON, Canada K1H 8M8; Newborn Screening Ontario, 415 Smyth Road, Ottawa, ON, Canada K1H 8M8; Newborn Screening Ontario, 415 Smyth Road, Ottawa, ON, Canada K1H 8M8; Department of Pediatrics, Windsor Regional Hospital, 1995 Lens Avenue, Windsor, ON, Canada N8W 1L9; Newborn Screening Ontario, 415 Smyth Road, Ottawa, ON, Canada K1H 8M8; Department of Pediatrics, Kingston Health Sciences Centre, 76 Stuart St, Kingston, ON, Canada K7L 2V7; Department of Pediatrics, Queens' University, Kingston, 76 Stuart St, Kingston, ON, Canada K7L 2V7; Newborn Screening Ontario, 415 Smyth Road, Ottawa, ON, Canada K1H 8M8; Department of Pathology and Laboratory Medicine, University of British Columbia, 6200 University Blvd, Vancouver, BC, Canada V6T 1Z4; Department of Pathology and Laboratory Medicine, British Columbia Children's Hospital, 4500 Oak St, Vancouver, BC, Canada V6H 3N1; Department of Pediatrics, Hospital for Sick Children, 555 University Avenue, Toronto, ON, Canada M5G 1X8; Department of Pediatrics, University of Toronto, 555 University Avenue, Toronto, ON, Canada M5G 1X8; Department of Pediatrics, Children's Hospital of Eastern Ontario, 401 Smyth Road, Ottawa, ON, Canada K1H 8L1; Department of Pediatrics, University of Ottawa, 75 Laurier Avenue East, Ottawa, ON, Canada K1N 6N5; Newborn Screening Ontario, 415 Smyth Road, Ottawa, ON, Canada K1H 8M8

**Keywords:** congenital adrenal hyperplasia (CAH), newborn screening, 21-deoxycortisol, steroid profile

## Abstract

**Purpose:**

Newborn screening for 21-hydroxylase deficiency congenital adrenal hyperplasia (CAH) has a high false-positive rate. A second-tier steroid profile using liquid chromatography mass spectrometry can improve specificity. Multiple screening algorithms were evaluated to optimize the performance of screening for salt-wasting CAH (SW-CAH).

**Methods:**

Principal components analysis guided potential combinations of steroid biomarkers for evaluation in a study population of 1710 immunoassay-positive samples proceeding to the second-tier steroid profile in the Newborn Screening Ontario program between August 2020 and April 2023. A Monte Carlo simulation was used to evaluate the performance of algorithms and cutoffs.

**Results:**

Optimal performance for the identification of SW-CAH used a 3-component second-tier algorithm: detectable 21-deoxycortisol (≥ 2.1 nmol/L); 17-hydroxyprogesterone + 21-deoxycortisol ≥ 40 nmol/L; and ratio of (17-hydroxyprogesterone + 21-deoxycortisol)/cortisol ≥ 0.3. All 8 cases of SW-CAH were accurately identified with a positive predictive value of 70% and 100% sensitivity for SW-CAH, whereas 1 known case of simple virilizing (SV) CAH screened negative. When applied to 26 historical cases, the algorithm identified all 13 cases of SW-CAH and all 6 SV-CAH cases, whereas other forms of CAH were filtered out because of low 21-deoxycortisol.

**Conclusion:**

Using 21-deoxycortisol for second-tier screening and applying a 3-component algorithm can improve performance of newborn screening for SW-CAH, reducing burden on patients and the health care system. Although cases of SV-CAH may be identified, the thresholds were set to identify life-threatening SW-CAH with a high positive predictive value and 100% sensitivity.

Congenital adrenal hyperplasia (CAH) is caused by the loss of enzyme activity required for adrenal steroidogenesis, the most common form being 21-hydroxylase deficiency (21-OHD). Of those with classical 21-OHD CAH, 75% have the more severe salt-wasting (SW) form, which can present with life-threatening adrenal crisis in the first few weeks of life because of a deficiency of both glucocorticoids and mineralocorticoids [1]. Those with simple virilizing (SV) CAH typically present with signs of excess androgens at birth or later in childhood. Many jurisdictions have introduced newborn screening (NBS) for CAH with a variety of screening algorithms that employ a first-tier immunoassay to measure 17-hydroxyprogesterone (17-OHP) in dried blood spot (DBS) samples, and in some programs, measurement of steroid hormones via liquid chromatography mass spectrometry (LC-MS/MS) is used as a second-tier screen [1]. The nomenclature has evolved to refer to SV- and SW-CAH as a single entity, classical CAH. However, we have retained the distinction in this study as NBS is focused on the more severe SW form of CAH.

The hormones most commonly quantified for second-tier CAH screening by LC-MS/MS have included 17-OHP, androstenedione (A4) and cortisol (F), and more recently, 21-deoxycortisol (21-DF) and 11-deoxycortisol (11-DC) (Figure 1). The use of steroid ratios can further improve screening metrics, with published ratios including: (17-OHP + androstenedione)/cortisol [2-5]; (17-OHP + 21-deoxycortisol)/cortisol [6]; and 17-OHP/11-deoxycortisol [7]. The concentration of 21-deoxycortisol is elevated in those with 21-OHD and low or undetectable in unaffected individuals [8]. Measuring 21-deoxycortisol has demonstrated excellent sensitivity and specificity for CAH screening [9-11]. The NBS program in the Netherlands performed a pilot study adding a second-tier 21-deoxycortisol to both positive and inconclusive first-screen positive DBS samples [11] and eliminated all false-positive cases. Held et al reported a positive predictive value (PPV) of 91.7% with 100% sensitivity using 21-deoxycortisol alone as a second-tier screen [10].

**Figure 1.**
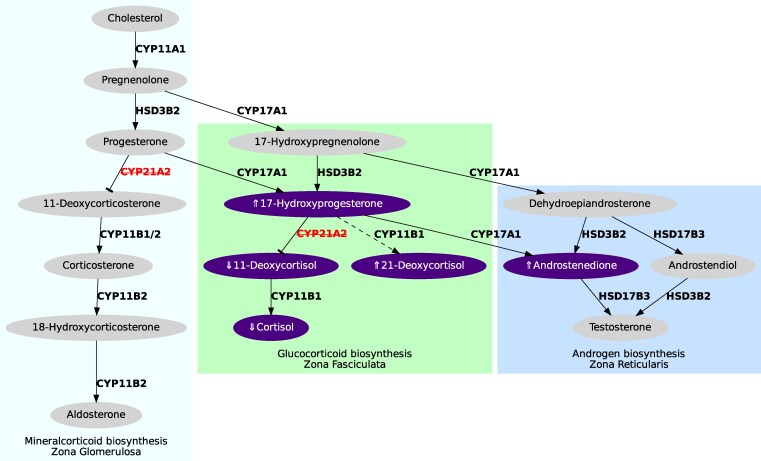
Adrenal steroidogenic pathway in 21-hydroxylase deficiency congenital adrenal hyperplasia. Steroids measured in the second tier of the proposed CAH screening algorithm are shown in bold. The enzymes and factors indicated: P450scc, cholesterol side-chain cleavage enzyme (*CYP11A1*); StAR steroidogenic acute regulatory protein; 3βHSD2, 3β-hydroxysteroid dehydrogenase type 2 (*HSD3B2*); P450c21, steroid 21-hydroxylase (*CYP21A2*); P450c11β, 11β-hydroxylase (*CYP11B1*); P450c11AS, aldosterone synthase (*CYP11B2*); P450c17, 17α-hydroxylase/17,20-lyase (*CYP17A1*); b5, cytochrome b5; 17βHSD3/5, 17β-hydroxysteroid dehydrogenase, type 3 or 5 (*HSD17B3*); P450c11β, (*CYP11B1*); DHEA dehydroepiandrostenedione.

In 2007, Newborn Screening Ontario (NSO), Canada, implemented NBS for CAH with a primary target of SW classical 21-OHD. First-tier screening measures 17-OHP by immunoassay with gestational age-based cutoffs. Since 2011, second-tier steroid profiling is performed on the same sample to measure LC-MS/MS 17-OHP levels and the ratio of (17-OHP + A4)/F. Despite the use of a 2-tiered, multianalyte screening approach, the PPV for SW-CAH screening in Ontario was low at 3.1%. In keeping with published reports [1], the highest rate of false-positive samples occurred in premature infants. The current study analyzed multiple combinations of steroid measurements, ratios, and thresholds with a goal of optimizing the PPV of screening for SW-CAH while retaining a high sensitivity.

## Materials and Methods

### Study Population

NSO screens approximately 145 000 newborns annually. CAH screening employs a first-tier 17-OHP immunoassay measurement with gestational age (GA)- and birthweight (BW)-dependent cutoffs [12]. A second-tier LC-MS/MS steroid panel based on 17-OHP concentration and the ratio of (17-OHP + A)/cortisol is then applied. Because of conservative first-tier thresholds selected to optimize sensitivity in this population, a relatively high proportion (0.4%) of samples proceed to second-tier steroid profiling, of which 54% are from premature infants < 37 weeks’ GA. The primary target condition is SW 21-CAH. Other diseases, including milder forms of 21-OHD (SV or nonclassical CAH) and other types of CAH (11 β-hydroxylase deficiency and 3-β dehydrogenase deficiency [3 BOHSD]) may be identified. Only the primary target of SW-CAH was used for PPV and sensitivity/specificity analysis. The diagnosis of SW-CAH was determined by the reporting clinician and supported by the presence of hyponatremia and hyperkalemia. NSO is a Public Health program that is universally available at no cost to participants, with 99.55% of newborns fully screened. NSO has a formal system for direct reporting of missed cases to NSO. In addition, a focused provider survey was sent to all pediatric endocrinologists practicing in the province of Ontario aimed at identifying any false-negative cases of classical CAH, either SW or SV.

For the population study, a dataset was created of all samples that progressed to second-tier screening between August 3, 2020, and April 4, 2023 (n = 1710). Historical samples positive for SW, SV, and nonclassical 21-OHD CAH as well as 3β-hydroxysteroid dehydrogenase deficiency and 11β-hydroxylase deficiency were used for the validation phase of the study. DBS samples for 26 selected screen positives from March 3, 2017, to October 18, 2022, stored at −80 °C were reanalyzed using the improved LC-MS/MS method. Repeat steroid analysis was compared against steroid analysis at the time of screening to ensure that sample degradation had not occurred.

### Analytical

First-tier screening for CAH requires measurement of 17-OHP using fluorometric immunoassay

(Revvity GSP). Screening results are primarily determined using gestation age-based 17-OHP thresholds [12]. If GA is unreported, BW-based 17-OHP thresholds are used instead. First-tier screening for SW-CAH employs an immunoassay for 17-OHP with gestational age and birth weight. DBS 17-OHP, 21-DF, 11-DC, androstenedione, and cortisol were quantified via LC-MS/MS. Steroids were extracted from a 3.2-mm punch of the DBS with 3/1 methanol/water containing internal standards. The solution was then dried under nitrogen gas and reconstituted using a 50/50 methanol/water solution with 0.1% formic acid. The steroids were resolved by LC on an Acquity HSS T3 1.8 µm, 2.1 × 50 column (Waters) using a methanol, water gradient (mobile phase A: 2-mM ammonium acetate + 0.1% formic acid in water, mobile phase B: 2-mM ammonium acetate + 0.1% formic acid in methanol). A Waters TQS-micro MS/MS, operating in positive ion mode with multiple reaction monitoring, was used for detection. Quantifying and qualifying multiple reaction monitoring transitions were adopted from Rossi et al [13]. The DBS calibrators were prepared using 50% hematocrit representing an estimated mean for newborns over a range of gestational ages.

The following improvements were made to the second tier LC-MS/MS steroid panel to improve traceability, long-term accuracy, and address analytical interferences: deuterated internals standards were replaced with carbon 13 labeled internal standards where available; certified reference materials (Cerilliant, TX, USA) were used for calibration; chromatography was modified to achieve baseline resolution between 21-DF ([M + H]+ = 347.3 Da) and isobars 11-DC, and corticosterone before multiple reaction monitoring for precursor and product ions. 17-OHP, androstenedione, and cortisol are identified based on distinct retention times and precursor/product ion pairs. In addition, quantifier and qualifier ions are used to monitor potential interferences with 17-OHP. Additional low-concentration 21-deoxycortisol calibrator points were added to the method to achieve a lower limit of detection (LoD) of 2.1 nmol/L [14]. The lower limit of quantitation for 21-deoxycortisol was 3.4 nmol/L. This study includes NBS samples analyzed between the implementation of this LC-MS/MS method on August 3, 2020, and April 28, 2023

### Data Analysis

**Figure 2.**
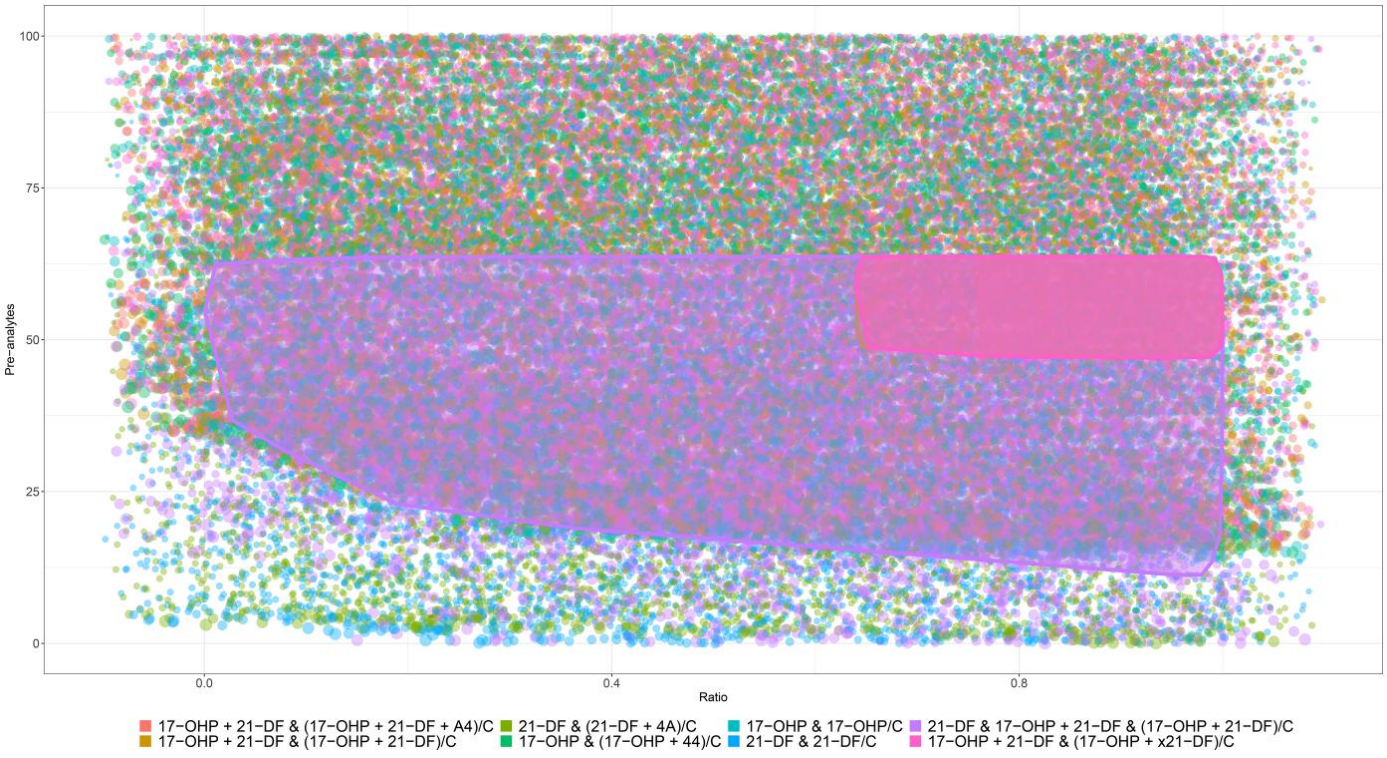
A Monte Carlo simulation of screening algorithms. Each point is a simulated screening approach using a combination of preanalytes (before the enzyme block) and steroid ratios. The color of the point is based on the algorithm used, the size of the point reflects the total number of screen positive cases, the position of the point is determined by the preanalyte and ratio cutoff values used in the algorithm. Regions with 100% sensitivity and ≥75% PPV for salt wasting 21-OHD CAH are delineated to indicate the in-use algorithm. The large purple region corresponds with proposed algorithm which was then selected for further evaluation.

Principal components analysis (PCA) was used to explore potential combinations of steroid biomarkers for evaluation. Candidate analytes included combinations of “pre” analytes that are proximal to the enzyme block (17-OHP, androstenedione, 21-deoxycortisol) and a ratio of these preanalytes to those beyond the block including cortisol and 11-deoxycortisol. The PCA results influenced which analytes were included in the evaluation. Eight potential screening algorithms were selected based on review of the steroid biosynthesis pathway, literature [2-11], and PCA. These are detailed in the legend of Fig. 2. A Monte Carlo simulation was used to evaluate the performance of each of these algorithms in the identification of SW-CAH. In the simulation, each of the 8 potential second-tier screening algorithms was applied to the population data 10 000 times. In each iteration of the simulation, random threshold values were selected from uniform distributions and applied to the population data to identify algorithms that could achieve a PPV of 75% while maintaining 100% sensitivity. All statistical analysis and data visualization was performed using R [15].

## Results

**Figure 3.**
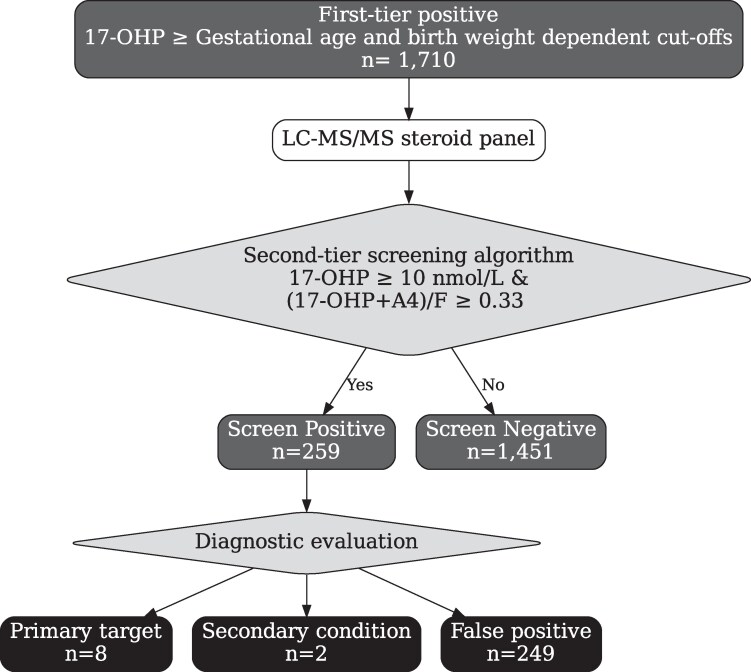
Screening disposition of the 1710 newborns positive on tier 1 screening (immunoassay 17-hydroxyprogesterone) and for whom tier 2 steroid results were available for inclusion in the population study.

This dataset contains 1710 specimens that were positive on the first-tier 17-OHP immunoassay and went on to second-tier steroid analysis via the improved LC-MS/MS method. Using the existing NSO second-tier algorithm, 259 were positive samples and 1451 were negative with no false negatives (Fig. 3). This algorithm identified all 8 cases of SW-CAH (primary target) and 3 with variant conditions (ie, 1 SV-CAH and two 3-BOHSD CAH). The mean age at diagnosis of cases was 5.25 days (range, 3-8 days). Using the existing second-tier screening algorithm employed by NSO, the PPV for SW-CAH was 3.1% with a sensitivity of 100%. The provider surveys identified no missed cases of SW-CAH.

**Figure 4.**
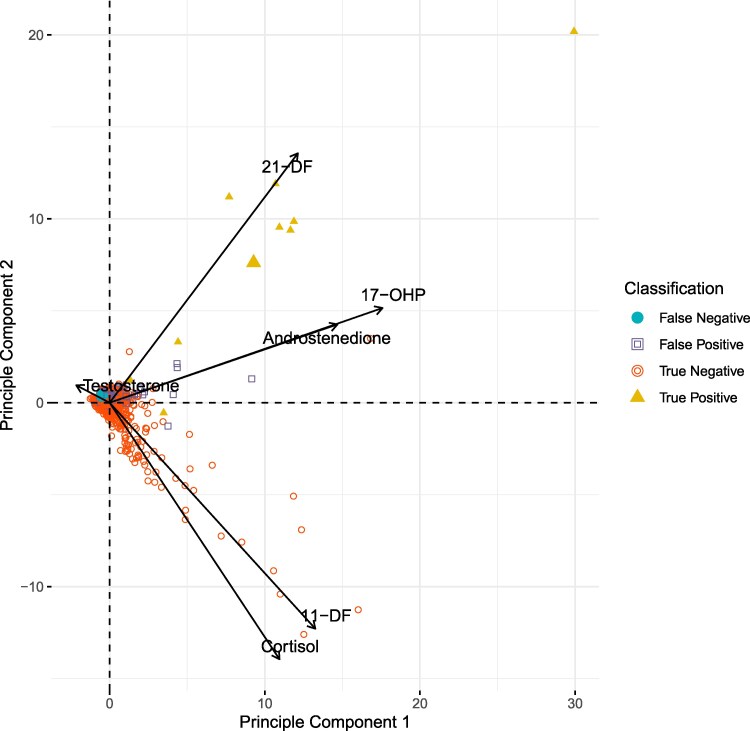
Principal component analysis (PCA) of dried blood spot steroid concentrations. PCA scores for true-positive (solid orange triangles), true-negative (open purple squares), and false-positive (solid teal circles) samples were plotted on principal components 1 and 2. Vectors represent the coefficients of the steroid concentrations used in PCA. The direction of the vectors indicates the correlation of that steroid concentration with the principal component axes.

The first 2 principal components in the PCA capture >60% of the variance in the data set. The relative length of the loading vectors in the biplot show the contribution of each steroid measurement to principal components 1 and 2. The angle of the loading vectors show that cortisol concentration is not correlated with 21-deoxycortisol concentration and that these steroids contribute to separation of positive and negative cases along principal component 2 (Fig. 4). 17-OHP and androstenedione vectors are collinear in the subject space indicating close correlation and therefore redundant information. Although variance in both 17-OHP and 21-DF contributes to separation of true-positive cases from unaffected newborns, they do not overlap, indicating that both variables contribute to separation of positive cases. One positive case with 3β-hydroxysteroid dehydrogenase deficiency and twins with SW-CAH cluster near the origin of the biplot, consistent with the need for a multivariate screening algorithm. Monte Carlo simulation was used to evaluate the performance of potential multivariate screening algorithms, identifying the strongest performance with a 3-component algorithm using 17-OHP, 21-deoxycortisol, and cortisol (Figure 2). The randomly assigned ratio and preblock (pre) thresholds for each simulated outcome were plotted; the purple region encompasses the range of threshold values for which the proposed algorithm maintains a PPV greater than 75% with 100% sensitivity for SW.

Levels of 21-deoxycortisol were significantly higher in confirmed cases of CAH than the unaffected newborns with a mean (SD) of 49 (28) nmol/L in affected and undetectable in 93% of unaffected neonates. Measuring 21-deoxycortisol alone and applying previously published thresholds to optimize sensitivity (≥2.5 nmol/L or 0.85 ng/mL) and specificity (≥6.56 nmol/L or ≥2.27 ng/dL) yielded a PPV of 5% and 73%, respectively, with a false-negative case using the higher threshold. In our study population a 21-deoxycortisol threshold of ≥4.6 nmol/L was required for 100% sensitivity, with a resulting PPV of 30%.

**Figure 5.**
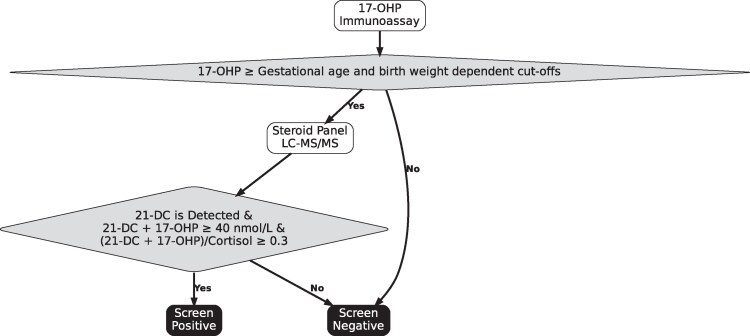
Screening algorithm for salt wasting 21-hydroxylase deficiency congenital adrenal hyperplasia.

**Figure 6.**
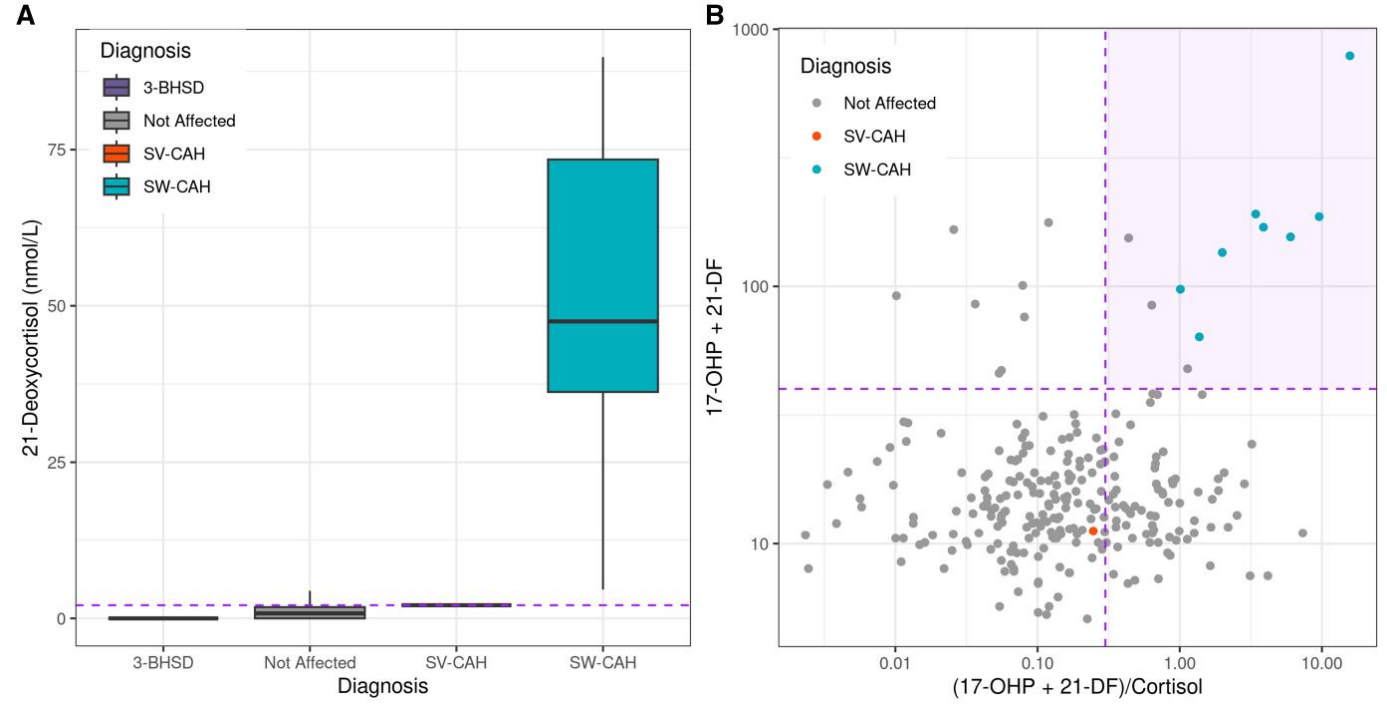
Analysis of the proposed second-tier algorithm applied to the population study data with sensitive and specific cutoff values. Proposed second-tier algorithm applied to the 1710 steroid profiles in the population study. (A) Boxplot of 21-deoxycortisol concentration definitive diagnosis after diagnostic testing, horizontal dashed purple line at the limit of detection 2.1 nmol/L. (B) Scatter plot of samples with quantifiable detectable 21-deoxycortisol (≥ 2.1 nmol/L). Dashed purple lines indicate cutoffs plotted on a logarithmic axis: 17-OHP + 21-deoxycortisol ≥ 40 and (17-OHP + 21-deoxycortisol)/cortisol ≥ 0.3.

Table 1 shows combinations of potential cutoffs for the 3 components that would yield PPVs of 50%, 70%, and 100% for SW-CAH. The intermediate cutoffs were selected to provide a safety margin. Applying the screening algorithm in Fig. 5, 100% sensitivity and 70% PPV for SW- CAH was achieved in this study population with thresholds of a detectable 21-deoxycortisol (LoD 2.1 nmol/L); 17-OHP + 21-deoxycortisol ≥ 40 nmol/L; and a ratio of (17-OHP + 21-deoxycortisol)/cortisol of ≥0.3 (Figure 6). The 3 false-positive cases were all in premature infants; 2 were 26 weeks’ and 1 was 33 weeks’ GA and all had borderline 21-DF levels at 2.1 to 2.2 nmol/L. Their 17-OHP values ranged from 45 to 152 nmol/L. Mean (SD) values for 21-DF, 17-OHP, and cortisol for true- and false-positive cases are shown in Table 2. In the original population sample, 170 of the 249 false positives (69%) were in neonates <37 weeks’ GA. Of the true-positive cases of SW-CAH, 2 were 36 weeks’ GA and the rest were full term. The PPV was 40% for premature and 100% for full-term infants. Applying the same cutoff irrespective of GA yielded an overall PPV of 70%.

**Table 1.** Performance metrics for Various cut offs for the prediction of salt wasting 21- hydroxylase deficiency congenital adrenal hyperplasia using a 3 component newborn screening algorithm

21 Deoxycortisol (21-DF) (nmol/L)	21-DF + 17 OHP (nmol/L)	[17-OHP ± 21-DF] Cortisol	PPV	NPV
Detectable (>2.1)	≥ 35	≥ 0.1	0.5	1
Detectable (>2.1)	≥ 40	≥ 0.3	0.70	1
≥ 3.4	≥ 40	≥ 0.4	1.0	1

Steroid concentrations are shown in nmol/L.

Abbreviations: 17-OHP, 17-hydroxy progesterone; 21-DF, 21-deoxycortisol; NPV, negative predictive value; PPV, positive predictive value.

**Table 2.** Summary statistics (mean (SD)) of laboratory values for screen-positive cases of congenital adrenal hyperplasia

Diagnosis	n	GA weeks	BW grams	21-DF	17-OHP	Cortisol
SW-CAH	8	37.9 (2.1)	2873 (738)	49.5 (29.7)	174.0 (215.8)	50.8 (24.1)
Not affected	3	28.6 (3.9)	1140 (395)	2.1 (0.1)	93.4 (53.9)	176.0 (159.7)

Steroid concentrations are shown in nmol/L.

Abbreviations: 17-OHP, 17-hydroxy progesterone; 21-DF, 21-deoxycortisol; BW, birthweight; GA, gestational age; SW-CAH, salt-wasting 21-hydroxylase deficiency congenital adrenal hyperplasia.

**Figure 7.**
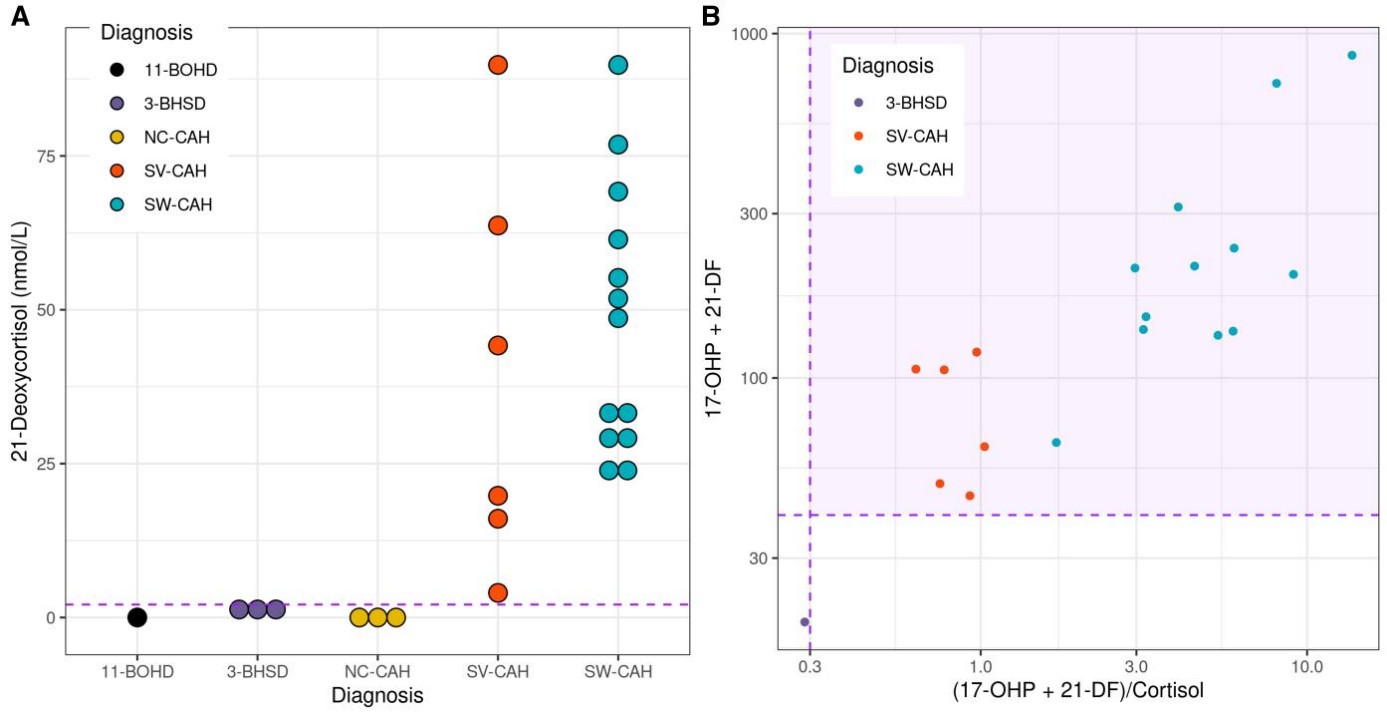
Analysis of proposed second-tier algorithm applied to historical screen-positive samples proposed second-tier algorithm applied to selected historical control samples with known diagnoses of salt wasting 21-OHD CAH and variant forms of CAH. (A) Boxplot of 21-deoxycortisol concentration grouped by definitive diagnosis after diagnostic testing, horizontal dashed purple line at the limit of detection 2.1 nmol/L. (B) Scatter plot of samples with quantifiable detectable 21-deoxycortisol (≥ 2.1 nmol/L). Dashed purple lines indicate cutoffs on a logarithmic scale: 17-OHP + 21-deoxycortisol ≥ 40 nmol/L and (17-OHP + 21-deoxycortisol)/cortisol ≥ 0.3.

Further testing of the proposed second-tier algorithm was applied to the DBS of a historical cohort of 26 newborns diagnosed with the primary target or variant conditions before the implementation of the improved DBS steroid LC-MS/MS method (Figure 7). All 13 cases of SW-CAH were identified in addition to all 6 cases of SV-CAH. The other forms of CAH were not identified using the proposed algorithm, having been filtered out based on 21-deoxycortisol levels. This included 1 case of 11-BOHD, 3 with 3-BOHSD, and 3 with nonclassical 21-OHD.

## Discussion

NBS for CAH using 17-OHP-based screening algorithms has a high rate of false-positive results, particularly in premature infants. Despite a growing body of evidence on the utility of 21-DC in NBS for CAH, this approach has not been widely adopted in NBS programs in North America [16]. This study demonstrates the effectiveness of inclusion of 21-DC in combination with 17-OHP and cortisol in a 3-component second-tier algorithm CAH. Importantly, steroid profiles were analyzed routinely as part of the existing CAH NBS algorithm to reflect a realistic analytical performance in a clinical setting.

Given the high false-positive rates on immunoassay screening [1, 17, 18], particularly in premature or unwell infants [19], most screening programs employ a first-tier screen with immunoassay quantification of 17-OHP using BW [20-22] or GA [12] stratification or a combination of both [23, 24]. GA-based stratification is recommended [1]. A change from BW to GA criteria by the Netherlands improved the PPV for CAH screening from 4.5% to 16% [12]. Finally, the addition of a second-tier steroid profile has been shown to reduce the rate of false positives [2-4, 6, 20, 25, 26].

The screening program in New South Wales, Australia [5], applied a 2-tier screening pathway with a first-tier immunoassay 17-OHP of > 22 nmol/L and/or top 2% of the daily assay, proceeding to LC-MS/MS for 17-OHP, androstenedione, and cortisol. Samples with a ratio of (17-OHP + A4)/cortisol >2 and 17-OHP > 200 nmol/L were considered as presumptive positive. Over a 2-year period, they identified 10 newborns with CAH (9 SW) with no known false negatives, yielding a PPV of 71.4%, sensitivity of 100%, and specificity of 99.9%. Using the same approach with more conservative thresholds, our current PPV is 3.1% for SW-CAH. These thresholds (17-OHP ≥ 10 nmol/L and ratio of ≥ 0.33) were designed to provide 100% sensitivity based on cases identified in our population before implementation of steroid profiling.

There is strong rationale and emerging evidence for the use of 21-deoxycortisol for CAH screening. In individuals unaffected by CAH, the pathway to production of 21-deoxycortisol is relatively inactive compared to the energetically favorable pathway to 11-deoxycortisol via 21-hydroxylase. As a result, 21-deoxycortisol levels are usually low or undetectable [6, 9, 11, 27]. In those with a deficiency of 21-OHD, the markedly elevated circulating 17-OHP is a substrate for 11β-hydroxylase and is hydroxylated to form 21-deoxycortisol resulting in a significant elevation of the metabolite (Fig. 1) [28]. One of the challenges with the use of 21-DF in CAH screening has been separation of steroid isomers for 21-DF such as 11-DC and corticosterone [29]. The inability to do this will result in poor screening performance. This was addressed in our methodology with chromatographic separation of 21-DF, 11-DC, and corticosterone.

Using 21-deoxycortisol alone as a second-tier screen has been reported with good results. Held et al evaluated a streamlined decision tree using only 21-DF with a cutoff of ≥ 2.5 nM yielding a 91.7% PPV for 21-OHD CAH screening [10]. The population in that study was not a generalized screening population, but rather was enriched with positive cases. The increased prevalence of CAH in the study population will increase the PPV. A total of 906 specimens were used for the analysis, including 851 (94%) collected from presumptively unaffected newborns and 55 (6%) collected from newborns with confirmed 21-OHD. All 55 confirmed cases of 21-OHD were evaluated by pediatric endocrinologists, who reported clinical features of either SW 21-OHD or SV 21-OHD, and abnormal diagnostic testing, including elevated serum 17-OHP. In our population, applying this strategy resulted in a PPV of 5% with 100% sensitivity. This difference may have been influenced by the low specificity of our first-tier algorithm. Stroek et al [11] reported that, adding a second-tier 21-DF measurement to both positive and inconclusive 17-OHP immunoassay results eliminated all false positives using a threshold of 21-DF ≥ 1 nmol/L. Watanabe et al [27] selected 17-OHP, 21-DF, (A4 + 17-OHP)/F, and 11DF/17-OHP as screening markers for evaluation in a population of 63 screen-positive newborns, 26 with classical CAH and 37 false positives. Of these, 15 were born before 37 weeks’ GA: 2 cases and 13 false positives. They proposed the inclusion of 21-DF and steroid ratios such as (A4 + 17-OHP)/F and 11DF/17-OHP as markers for CAH screening. The selected cutoff for 21-DF was 2.9 nmol/L.

The strategy used in selecting algorithms for stochastic simulation used “preanalytes” (hormones proximal to the block, including 17-OHP, 21 deoxycortisol, and androstenedione) either alone or in combination, and a ratio of “preanalytes” to cortisol or 11-deoxycortisol. We excluded 11-deoxycortisol as it had initially coeluted with corticosterone and we had just 1 year of stable data. The best performance was achieved with 17-OHP + 21-deoxycortisol as the preanalytes and the numerator of the ratio. Of the false positives, all were premature infants, and all had 21-deoxycortisol levels near the LoD at 2.1 to 2.2 nmol/L. Adding 21-deoxycortisol alone as a third component of the algorithm further improved the PPV. Although androstenedione is commonly included in SP ratios, in our analysis, androstenedione was highly correlated with 17-OHP in positive cases and did not provide additional information.

Prematurity and age of collection are both important considerations when interpreting NBS screening results for CAH. Although 17-OHP is generally elevated in premature infants, Janzen et al found that 21-deoxycortisol is not [6], hence the potential to reduce the high false-positive rates of 17-OHP-based NBS programs in premature neonates. Held et al showed that, although 17-OHP and 21-deoxycortisol are both influenced by gestational age, the degree of impact is much greater on 17-OHP, and that 21-deoxycortisol was a better predictor of 21-OHD status. Watanabe et al found that the PPV did not differ between full-term and preterm neonates for the selected markers, except for 17-OHP [27]. While the 3 false-positive cases in our study were in premature infants, the number of false-positive retrievals for preterms with the inclusion of 21-DC was markedly reduced from 174 to 3. The PPV for preterm neonates remained high at 40% as compared to 100% for full term. Sample size was too small to evaluate cutoffs by gestational age. Applying the same cutoff regardless of GA yielded a PPV of 70%. The 2 cases of CAH in nonterm neonates were late premature infants at 36 weeks’ GA [10]. Held et al also found that the timing of collection did not influence 21-deoxycortisol levels [10] in a cohort without CAH. Watanabe et al showed that 21-DF levels in unaffected neonates [27] remain extremely low over the first week of life, regardless of GA [27]. The 21-DF trend over time is less clear for those affected with CAH. In our program, 98.5% of DBS collections occur between 24 and 48 hours; hence, the performance of 21-DF vs 17-OHP in the early neonatal period is most relevant.

The small number of primary screening targets is a limitation of the study. The data presented represent modeling and analysis of a retrospective cohort of infants who screened positive for CAH in Ontario since 2020 with just 8 cases of confirmed SW-CAH. To address the small number of CAH cases in our population, an additional 26 confirmed CAH cases diagnosed before 2018 were included as a validation set to test the performance of the algorithm on samples for cases of both SW-CAH and variant conditions. These DBS samples had been stored at −80 °C to reduce sample degradation. The target condition for NBS is severe SW-CAH and thresholds were chosen accordingly.

Applying this new algorithm to the study population would have reduced the number of patient retrievals by 96% from 259 to 11 with all cases of SW-CAH identified. An important goal of NBS is early diagnosis of neonates with potentially life-threatening, treatable conditions, while minimizing harms to unaffected infants. As such, the thresholds selected for this second-tier CAH screening algorithm were designed to optimize identification of infants with the life-threatening form, SW-CAH, with 100% sensitivity. The proposed algorithm yielded a PPV of 70% for the identification of SW 21-CAH in this limited population. Of note, the secondary causes of CAH such as 3 BOHSD and 11-BOH deficiency were not detected using this screening logic due to low 21-deoxycortisol levels in these conditions. These had been incidentally identified using the previous disease screening logic.

The 1 known case of SV-CAH during the population study was not identified using the new algorithm. This is not a primary target of screening, and reducing the thresholds to capture SV-CAH would negatively impact the PPV and result in many more false-positive retrievals, often preterm and critically ill newborns, while not changing the sensitivity to identify the target condition of life-threatening SW-CAH. The algorithm did detect SV-CAH in all 6 cases of SV-CAH in the historical validation phase, indicating that detection is possible. A larger population sample is needed to evaluate the sensitivity for identifying SV-CAH. It is unlikely that the NBS 21-DF thresholds will identify nonclassical 21OHD CAH, with reported basal levels of 21-DF in this condition being below the limit of detection at an average of 0.31 nmol/L [30]. It is important that clinicians are aware that a normal newborn screen does not rule out atypical, nonclassical, and non-SW classical forms of CAH and to investigate clinically suspected cases accordingly.

A limitation of the study is the absence of genotyping information on all cases of CAH to facilitate the distinction of SW- and SV-CAH. The diagnosis is based on the declaration of the treating physician in addition to biochemical evidence of hyponatremia, hyperkalemia, and when available, renin levels and molecular results. An expert panel discussed borderline cases for a consensus diagnosis of SW- vs SV-CAH, with additional information obtained from the treating endocrinologist as required. Molecular confirmation was available for 7 of the 8 cases of SW-CAH and none with SV-CAH. Our sensitivity for the target condition of SW-CAH is reported as 100%. We are unable to comment on the sensitivity to detect SV-CAH because our reporting system is focused on the life-threatening, SW form. We have a small community of pediatric endocrinologists and 5 tertiary care centers in Ontario. The 2 ongoing strategies to identify false-negative cases includes a formal reporting system, and regular (2-4 per year) meetings of the endocrinology disease-specific working group providing a forum for discussion of interesting and missed cases. This is open to all endocrinologists and has regular representation from each of the tertiary centers. For the purposes of the current study, a focused survey was sent to all pediatric endocrinologists in the province, which did not identify any missed cases of either SW- or SV-CAH.

## Conclusion

The addition of 21-deoxycortisol to second-tier screening and applying a 3-component algorithm improved the performance of screening for SW-CAH through NSO, identifying all cases of SW-CAH and yielding a PPV of 70%. Reducing the number of false-positive screening tests has the potential to lessen the burden on newborns and their families, newborn nurseries, diagnostic laboratories, and health care professionals involved in follow-up of positive NBS results. Optimal performance was achieved when DBS results met the following 3 criteria: detectable 21-deoxycortisol (LoD 2.1 nmol/L), 17-OHP + 21-deoxycortisol ≥ 40 nmol/L, and a ratio of (17-OHP + 21-deoxycortisol)/cortisol ≥ 0.3. Education is important to ensure that clinicians are aware that the less common and less severe forms of CAH are unlikely to be identified using this screening strategy because of low levels of 21-deoxycortisol in these variant forms. Evaluation of this proposed algorithm on a larger population is needed to confirm the performance metrics, including genotyping and identification of cases of classical SV-CAH.

## Data Availability

Restrictions apply to the availability of some or all data generated or analyzed during this study to preserve patient confidentiality or because they were used under license. The corresponding author will on request detail the restrictions and any conditions under which access to some data may be provide.

## Abbreviations

3 BOHSD: 3-β dehydrogenase deficiency
11-DC: 11-deoxycortisol
17-OHP: 17-hydroxy progesterone
21-DF: 21-deoxycortisol
21-OHD: 21-hydroxylase deficiency
A4: androstenedione
BW: birthweight
CAH: congenital adrenal hyperplasia
DBS: dried blood spot
F: cortisol
GA: gestational age
LC-MS/MS: liquid chromatography mass spectrometry
LoD: limit of detection
NBS: newborn screening
NSO: Newborn Screening Ontario
PCA: principal component analysis
PPV: positive predictive value
SV: simple virilizing
SW: salt wasting
